# A Case of Chronic Neutrophilic Leukemia Incidentally Detected by ^18^F-FDG PET/CT

**DOI:** 10.3390/diagnostics11040654

**Published:** 2021-04-05

**Authors:** Miju Cheon, Jang Yoo, Hae Su Kim, Eunsin Bae

**Affiliations:** 1 Department of Nuclear Medicine, Veterans Health Service Medical Center, 61 Jinhwangdoro-gil 53, Gangdong-gu, Seoul 05368, Korea; jang8214.yoo@gmail.com; 2 Division of Hematology and Oncology, Department of Internal Medicine, Veterans Health Service Medical Center, 61 Jinhwangdoro-gil 53, Gangdong-gu, Seoul 05368, Korea; cygnus@bohun.or.kr; 3 Division of Laboratory Medicine, Veterans Health Service Medical Center, 61 Jinhwangdoro-gil 53, Gangdong-gu, Seoul 05368, Korea; aidi99@bohun.or.kr

**Keywords:** chronic neutrophilic leukemia, neutrophilia, F-18 FDG PET, PET/CT, leukemia

## Abstract

Chronic neutrophilic leukemia (CNL) is a rare, potentially aggressive, myeloproliferative neoplasm. To the best of our knowledge, there are no previous reports dealing with ^18^F-FDG PET findings in CNL. We describe a case of CNL in a 69-year-old male, imaged with ^18^F-FDG PET/CT at diagnosis and during treatment.

A 69-year-old male presented with cough, sputum, and fatigue. Laboratory analysis showed marked leukocytosis, with a leukocyte count of 139.37 × 10^3^/μL. The differential count revealed 93.6% segmented neutrophils. A bone marrow aspirate smear revealed hypercellularity with 100% neutrophilic proliferation. An ^18^F-FDG PET/CT scan was performed to exclude the possibility of other malignancies. ^18^F-FDG PET/CT images were acquired 1 h after intravenous injection of 238 MBq of ^18^F-FDG. The PET/CT images showed a marked increase in FDG uptake in the bone marrow spaces. Additionally, there was an inflammatory lesion in the right lung (Figure 1). Except for the pulmonary inflammatory lesion, the patient had no suspected infection, no other malignant lesions, and no history of treatment with granulocyte colony-stimulating factor or erythropoietin. Therefore, we considered some types of leukemia or aggressive lymphoma. It is well known from some earlier studies that the neutrophil count is significantly correlated with bone marrow FDG uptake [1]. Mutation analysis for BCR-ABL1 and JAK2V617F was negative. Subsequent mutation analysis for CFS3R, a biomarker for chronic neutrophilic leukemia (CNL) diagnosis, was positive. Thus, a diagnosis for CNL was established. CNL is a rare, potentially aggressive, myeloproliferative neoplasm, for which current WHO diagnostic criteria include leukocytosis of ≥25 × 10^9^/L, of which ≥80% are neutrophils, with <10% circulating neutrophil precursors and with blasts rarely observed [2]. The patient was treated with hydroxyurea and ruxolitinib. The patient’s neutrophil count showed a decreasing trend in response to treatment and then suddenly increased dramatically. Therefore, P^18^F-FDG ET/CT was performed once more to exclude the possibility of the extra-bone marrow’s involvement in organs such as the spleen or lymph nodes. Despite the treatment, the neutrophil count continued to increase, and the patient died a few days later. To the best of our knowledge, this is the first report of intense FDG uptake in CNL.

## Figures and Tables

**Figure 1:**
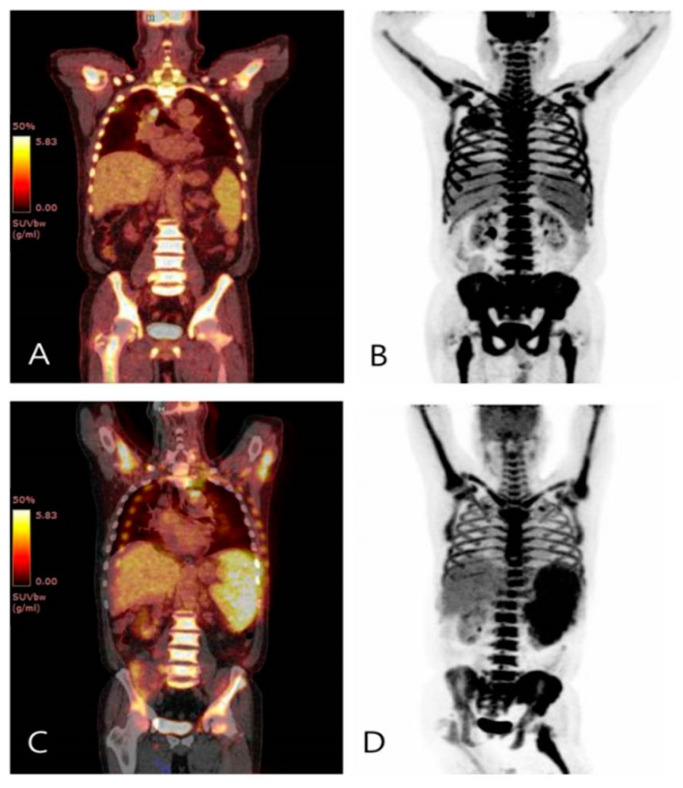
Coronal (**A**) and maximum-intensity projection (MIP) (**B**) F-18 FDG PET/CT images showed marked increase in FDG uptake in the bone marrow spaces (SUVmax 10.36). Additionally, abnormal FDG uptake in peribronchial consolidation of right lung suggests active inflammatory lesion. No other significant increase in FDG uptake suggesting malignancy was detected. A further increase in metabolism was demonstrated on coronal (**C**) and MIP (**D**) PET/CT images 3 months after the initiation of treatment, with SUVmax 12.17 for bone marrow and 7.17 for spleen.
